# Phosphatidylcholine as a potential AD‐related biomarker in Down syndrome, but not in Alzheimer's disease patients: Preliminary findings

**DOI:** 10.1002/alz70856_102841

**Published:** 2025-12-24

**Authors:** Bruna Berribilli Bortoleto, Tamires Alves Sarno, Leda Leme Talib, Wagner Farid Gattaz, Orestes Vicente Forlenza

**Affiliations:** ^1^ Laboratory of Neuroscience (LIM‐27), Institute of Psychiatry, Faculty of Medicine University of São Paulo, São paulo, São Paulo, Brazil; ^2^ Laboratory of Neuroscience (LIM‐27), Institute of Psychiatry, Faculty of Medicine University of São Paulo, Sao Paulo, São Paulo, Brazil; ^3^ Laboratory of Neurosciences (LIM27), Departamento e Instituto de Psiquiatria, Hospital das Clínicas, Faculdade de Medicina da Universidade de São Paulo, Sao Paulo, Sao Paulo, Brazil; ^4^ Laboratory of Neurosciences (LIM27), Departamento e Instituto de Psiquiatria, Hospital das Clínicas, Faculdade de Medicina da Universidade de São Paulo, São Paulo, São Paulo, Brazil

## Abstract

**Background:**

Individuals with Down Syndrome (DS) exhibit dementia‐related aging associated with the development of Alzheimer's Disease (AD). This relationship occurs because the amyloid precursor protein (APP), which plays a crucial role in AD pathogenesis, is located on chromosome 21—the same chromosome affected by trisomy in DS. Consequently, the overexpression of the APP gene in DS patients leads to the formation of beta‐amyloid plaques in the central nervous system, a hallmark of AD. Furthermore, recent studies conducted by other groups and ours suggest abnormalities in the lipidomics of AD patients, particularly in the analysis of lipid metabolites in plasma. The present study evaluates the potential use of phosphatidylcholine as an AD‐related biomarker in DS.

**Method:**

47 blood plasma samples were collected from the following groups: individuals with Alzheimer's disease (AD), Down syndrome individuals with cognitive decline due to Alzheimer's disease (DScd), Down syndrome individuals without cognitive decline due to Alzheimer's disease (DS), elderly cognitively healthy individuals (ECH), and young cognitively healthy individuals (YCH). High‐Pressure Liquid Chromatography coupled with Mass Spectrometry (HPLC‐MS/MS) was used for lipidomic analysis.

**Result:**

A total of 64 plasma phosphatidylcholines were identified in the groups by HPLC‐MS/MS. Principal Component Analysis (PCA) showed a PC1 of 51.7% and a PC2 of 16.2%, indicating that both account for the majority of the variance in the sample. Linear Discriminant Analysis (LDA) was able to discriminate the groups based on the specific phosphatidylcholine levels characteristic of each. Compared to euploid controls (elderly and young cognitively healthy individuals), Kruskal‐Wallis analysis showed that phosphatidylcholine 42:0 (PC 42:0) and phosphatidylcholine 42:1 (PC 42:1) (*p* < 0.040 and *p* <0.009, respectively) were elevated in the plasma of DScd individuals but not in Alzheimer's disease patients.

**Conclusion:**

Our preliminary results showed that DScd individuals presented elevated PC42:0 and PC42:1 compared to cognitively healthy controls. These findings suggest a distinct lipidomic profile that may serve as a biomarker to differentiate the progression of dementia in DS from Alzheimer's disease patients.

